# Vascular Dysfunction in Horses with Endocrinopathic Laminitis

**DOI:** 10.1371/journal.pone.0163815

**Published:** 2016-09-29

**Authors:** Ruth A. Morgan, John A. Keen, Brian R. Walker, Patrick W. F. Hadoke

**Affiliations:** 1 University/BHF Centre for Cardiovascular Science, The Queen’s Medical Research Institute, University of Edinburgh, Edinburgh, United Kingdom; 2 Royal (Dick) School of Veterinary Studies, University of Edinburgh, Midlothian, United Kingdom; University of Bristol, UNITED KINGDOM

## Abstract

Endocrinopathic laminitis (EL) is a vascular condition of the equine hoof resulting in severe lameness with both welfare and economic implications. EL occurs in association with equine metabolic syndrome and equine Cushing’s disease. Vascular dysfunction, most commonly due to endothelial dysfunction, is associated with cardiovascular risk in people with metabolic syndrome and Cushing’s syndrome. We tested the hypothesis that horses with EL have vascular, specifically endothelial, dysfunction. Healthy horses (n = 6) and horses with EL (n = 6) destined for euthanasia were recruited. We studied vessels from the hooves (laminar artery, laminar vein) and the facial skin (facial skin arteries) by small vessel wire myography. The response to vasoconstrictors phenylephrine (10^−9^–10^-5^M) and 5-hydroxytryptamine (5HT; 10^−9^–10^-5^M) and the vasodilator acetylcholine (10^−9^–10^-5^M) was determined. In comparison with healthy controls, acetylcholine-induced relaxation was dramatically reduced in all intact vessels from horses with EL (% relaxation of healthy laminar arteries 323.5 ± 94.1% v EL 90.8 ± 4.4%, P = 0.01, laminar veins 129.4 ± 14.8% v EL 71.2 ± 4.1%, P = 0.005 and facial skin arteries 182.0 ± 40.7% v EL 91.4 ± 4.5%, P = 0.01). In addition, contractile responses to phenylephrine and 5HT were increased in intact laminar veins from horses with EL compared with healthy horses; these differences were endothelium-independent. Sensitivity to phenylephrine was reduced in intact laminar arteries (P = 0.006) and veins (P = 0.009) from horses with EL. Horses with EL exhibit significant vascular dysfunction in laminar vessels and in facial skin arteries. The systemic nature of the abnormalities suggest this dysfunction is associated with the underlying endocrinopathy and not local changes to the hoof.

## Introduction

Laminitis is a crippling disease of the horse’s hoof resulting from mechanical failure of the laminar tissue, an interdigitating fibrous structure which suspends the distal phalanx within the hoof capsule. Failure of this tissue results in rotation and sinking of the distal phalanx causing acute pain when the horse bears weight. This condition is of huge clinical significance affecting up to 34% of horses [1] and commonly necessitates euthanasia on welfare grounds. Up to 90% of laminitis cases occur in association with the common conditions of Equine Cushing’s Disease (Pituitary Pars Intermedia Dysfunction, PPID) or Equine Metabolic Syndrome (EMS); so called endocrinopathic laminitis (EL) [2]. The causal mechanisms that link these endocrine disturbances with laminitis are unknown but since dysregulation of the blood flow to the laminae in both acute sepsis-related laminitis [3] and in chronic forms [4, 5] has been demonstrated, vascular dysfunction may represent a feasible link.

Human endocrine disturbances, such as Cushing’s syndrome and metabolic syndrome, are associated with systemic vascular dysfunction contributing to increased cardiovascular risk in these patients [6–8]. Endothelial dysfunction, manifest most commonly by failure of endothelial-dependent vasodilation, is a common finding in patients with atherosclerosis secondary to these conditions [9]. Given the complexities of these endocrine disturbances, it is difficult to determine causality particularly when likely candidates for altering vascular function such as insulin resistance, hyperinsulinaemia, cortisol dysregulation, inflammation and obesity often occur simultaneously [10]. Endothelial dysfunction is the most common vascular dysfunction associated with hyperinsulinaemia and insulin resistance in humans [11]. Insulin resistance is, independent of other risk factors, correlated with cardiovascular risk in humans [12, 13], as is obesity, specifically visceral body fat content [14] [15, 16]. In healthy human patients glucocorticoids inhibit cholinergic vasodilation [17] and potentiate the action of vasoconstrictors [18]. Endogenous or exogenous hypercortisolaemia is associated with endothelial dysfunction and increased risk of cardiovascular disease in human patients [19] [20] [21]. Numerous rodent, rabbit and canine models of diabetes, obesity and Cushing’s syndrome have been used to demonstrate endothelial dysfunction but there are little data on naturally occurring endocrine disease in other species [22, 23]. Pituitary Pars Intermedia Dysfunction in horses, is due to adenomatous or hyperplastic dysfunction of the pars intermedia of the pituitary and unlike other species is not associated with hypercortisolaemia.

As in humans, horses with metabolic syndrome or PPID often have multiple risk factors that are associated with endothelial dysfunction in humans. Insulin resistance and hyperinsulinaemia occur commonly in horses with EMS and PPID [24]. Horses with PPID are more likely to have laminitis if they are hyperinsulinaemic [25] and hyperinsulinaemia is a poor prognostic indicator for recovery from laminitis [26]. There is evidence that high dose insulin infusion can induce laminitis in otherwise healthy horses but the vascular function of these horses have not been investigated [27]. Generalised and regional adiposity and weight gain are independent predictors of laminitis risk [28, 29]. Exogenous glucocorticoid administration is, anecdotally, associated with the development of laminitis though convincing data of a causal relationship is lacking [30]. Cortisol has been shown to potentiate the response to vasoconstrictors in *ex vivo* studies of horses [31]. Treatments for laminitis have often empirically targeted a presumed vascular dysfunction with limited success [32]. There is a critical need to determine if vascular dysfunction occurs in these horses so that our understanding of this disease can be enhanced.

In this study we aimed to characterise the function of small resistance vessels of the hoof, as well as remote systemic arteries of the facial skin, in healthy horses and those with EL. Though much of the human and rodent work investigating vascular function focusses on arteries, there is some evidence that, in laminitis, the laminar veins are differentially affected [33]. For this reason, we chose to include laminar veins in our study. Given that there are recognised differences between vascular beds in response to vasoactive mediators we chose to compare remote arteries of the facial skin.

In this study, therefore, we address the hypothesis that vascular dysfunction, specifically endothelial dysfunction, is a feature of EL and is not confined just to the vessels of the hoof.

## Materials and Methods

### Animals

This study was approved by the University of Edinburgh Veterinary Ethics and Research Committee (VERC 7014). Horses with chronic laminitis due to metabolic syndrome (EMS) or pituitary pars intermedia dysfunction (PPID), and healthy controls, destined for euthanasia, were recruited from clinics at the Royal (Dick) School of Veterinary Studies. All groups included females and castrated males, reflecting the clinical population in the UK. The age, breed, sex, body condition score (out of 5, a measure of obesity) [34], clinical features of previous laminitis (abnormal hoof growth) and medical history (specifically history of laminitis and glucocorticoid administration) were recorded. Blood was obtained after overnight fasting, between 0900h and 1100h, via an intravenous cannula inserted in the jugular vein for the purpose of euthanasia. ACTH, cortisol, and insulin concentrations were measured by chemiluminescent immunoassays validated for clinical use in the horse (Immulite 2000, Siemens, Camberley, UK) [35, 36]. Plasma triglycerides and glucose were measured using a colorimetric method based on the modified Jaffe’s reaction (IL650 analyser, Instrumentation Laboratories, Barcelona, Spain).

Horses were humanely euthanased with intravenous quinalbarbitone sodium and cinchocaine hydrochloride (1mL/10Kg bodyweight; Somulose, Dechra Veterinary Products, Shrewsbury, UK).

### Tissue Preparation

Immediately following euthanasia tissue was harvested from the dorsal hoof and facial skin. The tissue was kept in physiological saline solution (PSS) at 4°C during dissection. Subcutaneous facial skin arteries (50–100μm in diameter) and laminar arteries and veins (100–500μm in diameter) were dissected from the tissue [37].

### Small Vessel Wire Myography

The vessels were divided into 2mm sections and stored overnight in PSS. Vessels were mounted on intraluminal wires in myography baths containing PSS maintained at 37°C and perfused with 95% O_2_/5%CO_2_ [37]. A minimum of 4 sections of each vessel were collected from each horse. Once mounted two sections of laminar arteries, laminar veins and facial arteries from each horse had the endothelium removed by rubbing the luminal surface with a small wire. Each vessel underwent an equilibration period at an initial tension of 4 mN for arteries and 2 mN for veins for 1 hour [37]. Vessel viability was assessed using three consecutive stimulations with 125 mM potassium physiological saline solution (KPSS) followed by a washout period. Cumulative concentration-response curves for each vessel were then obtained for phenylephrine (1x10^-9^ – 1x10^-4^ M) and 5-hydroxytryptamine (5HT) (1x10^-9^ – 1x10^-4^ M). All drugs were dissolved in PSS. Following contraction with sufficient phenylephrine to produce 80% of the KPSS response (3x 10^−7^ M), a cumulative concentration-response curve was obtained for acetylcholine (1x10^-9^ – 1x10^-4^ M).

### Statistical Analysis

Data reported are mean ± SEM. Contractions are expressed as force per unit length (mN/mm) and as a percentage of maximum KPSS-induced contraction, the maximal contraction (Emax) is reported for each agonist. Relaxation is expressed as percentage of pre-contraction with phenylephrine. Results from the two sections of each vessel type were averaged for further analysis. Sigmoid curves were fitted to the cumulative concentration-response data, 4 parameter logistic modelling performed. Data were tested for normality using a Kolmogorov-Smirnov test. Sensitivity to agonists is expressed as -log EC_50_ (pD_2_) for constrictors or -log IC_50_ for acetylcholine. The curves were analysed by 2-way ANOVA and post-hoc Tukey test. Comparisons of the group means was performed using Student’s unpaired t-test or Mann-Whitney U test following normality testing. Graphpad Prism 4 and SPSS were used for all statistical testing. Significance was defined at P<0.05.

## Results

### Animals

Six healthy horses, with no history of laminitis and 6 horses with chronic EL (4 with EMS and 2 with PPID) were included in the study. The healthy group consisted of 4 castrated males and 2 non-pregnant females. The EL group consisted of 3 castrated males and 3 non-pregnant females. Horses in the EL group were not suffering from acute laminitis at the time of euthanasia. None of the animals had a history of glucocorticoid administration in the previous month. Clinical and biochemical characteristics are given in Table 1. Horses with EL had higher resting serum insulin and triglyceride concentrations and high body condition scores (obesity) compared with healthy horses.

**Table 1:** Clinical and biochemical characteristics of the study groups; healthy and those with endocrinopathic laminitis (EL).

	Healthy	EL	P Value
**Age (years)**	19.1 ± 1.4	19.3 ± 1.4	0.46
**Body Condition Score (/5)**	2.0 ± 0.2	3.9 ± 0.2	<0.001
**Fasting Insulin (mIU/L)**	4.1 ± 1.4	57.1 ± 29.0	0.01
**Glucose (mmol/L)**	5.2 ± 0.2	4.9 ± 0.3	0.28
**Triglycerides (mmol/L)**	0.29 ± 0.02	0.54 ± 0.08	0.006
**Cortisol (nmol/L)**	150.6 ± 14.1	120.4 ± 12.3	0.35
**ACTH (pg/mL)**	27.6 ± 11.5	115.1 ± 54.3	0.001

Biochemical analyses were performed on fasted morning serum/plasma samples. Data are mean ± SEM. Following a Kolmogorov-Smirnov test for normality, comparisons between groups were by Student’s t-test or Mann-Whitney U test.

### Response of intact vessels from healthy horses to vasoconstrictors

**Fig 1:**
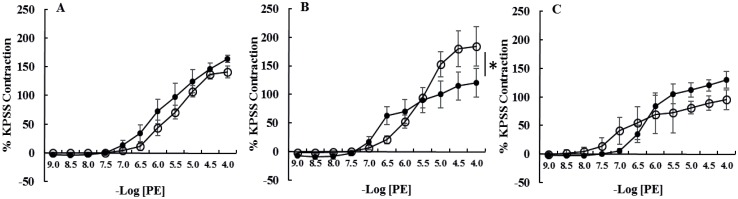
Cumulative concentration response curves to phenylephrine (PE) of intact [A] laminar arteries, [B] laminar veins and [C] facial skin arteries from healthy horses (●, n = 6) and horses with endocrinopathic laminitis (○, n = 6). Data are expressed as a percentage of maximal contraction in response to high potassium saline (KPSS). Data are mean ± SEM. Curves were compared using two-way ANOVA. *P<0.05. The veins from horses with EL had larger maximal contractions in response to PE compared to healthy horses.

**Fig 2:**
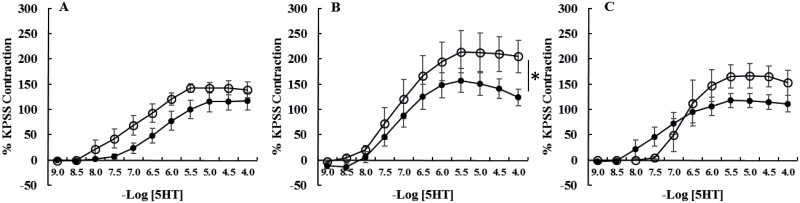
Cumulative concentration response curves to 5-hydroxytryptamine (5HT) of intact [A] laminar arteries, [B] laminar veins and [C] facial skin arteries from healthy horses (●, n = 6) and horses with endocrinopathic laminitis (○, n = 6). Data are expressed as a percentage of maximal contraction in response to high potassium saline (KPSS). Data are mean ± SEM. Curves were compared using two-way ANOVA. *P<0.05. The veins from horses with EL had larger maximal contractions in response to 5HT compared to healthy horses.

Laminar arteries and veins and facial arteries from healthy horses contracted in response to KPSS, phenylephrine and 5HT (Table 2, Figs 1 and 2). Laminar arteries produced a greater contraction than facial skin arteries which produced a much greater contraction than laminar veins for all vasoconstrictors (Table 2). Laminar veins were more sensitive to 5HT than either laminar arteries or facial skin arteries (P = 0.02), this was endothelium-dependent.

**Table 2:** Response of intact vessels from healthy horses and horses with endocrinopathic laminitis (EL) to vasoconstrictors.

		Intact laminar arteries		Intact laminar veins		Intact facial skin arteries	
		Healthy	EL	Healthy	EL	Healthy	EL
Number		6	6	6	6	6	6
**KPSS**	**Emax mN/mm**	31.4 ± 5.7	43.7 ± 6.9	5.9 ± 1.9	7.0 ± 1.8	20.2 ± 2.9	18.6 ± 3.4
**PE**	**Emax mN/mm**	49.8 ± 8.5	56.1 ± 8.7	6.4 ± 1.4	12.9 ± 2.1**	27.3 ± 5.7	17.1 ± 1.8
	**-Log EC**_**50**_ **(pD**_**2**_**)**	6.4 ± 0.1	5.6 ± 0.2*	6.8 ± 0.1	5.7 ± 0.3*	6.2 ± 0.2	6.5 ± 0.4
**5HT**	**Emax mN/mm**	34.2 ± 11.4	54.4 ± 8.8	8.1 ± 1.4	14.3 ± 2.1**	24.1 ± 5.5	27.8 ± 1.3
	**-Log EC**_**50**_ **(pD**_**2**_**)**	6.5 ± 0.2	6.1 ± 0.5	7.3 ± 0.1	7.4 ± 0.2	6.9 ± 0.2	7.0 ± 0.1

Responses to high potassium saline (KPSS), phenylephrine (PE) and 5-hyroxytryptamine (5HT) are given as absolute values (Emax mN/mm). Sensitivity is expressed as—Log EC_50_ (pD2). Data are mean ± SEM. Healthy v EL responses were compared by Student’s t-test or Mann Whitney U.

* denotes P<0.05 and

** denotes P<0.005 compared with healthy horses.

### Response of intact vessels from healthy horses to acetylcholine

**Fig 3:**
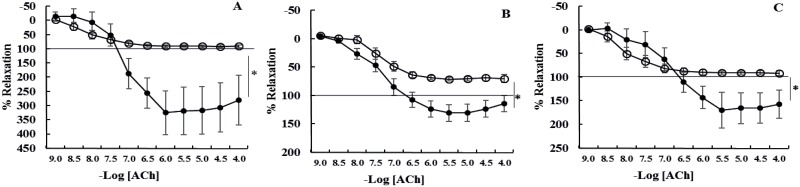
Cumulative concentration response curves to acetylcholine (ACh) of intact [A] laminar arteries, [B] laminar veins and [C] facial skin arteries from healthy horses (●, n = 6) and horses with endocrinopathic laminitis (○, n = 6). The horizontal line indicates baseline values prior to contraction with phenylephrine. Data are expressed as a percentage of maximal contraction in response to high potassium saline (KPSS). Data are mean ± SEM. Curves were compared using two-way ANOVA. *P<0.05, **P<0.005. Laminar arteries, veins and facial skin arteries of horses with EL relaxed significantly less than those from healthy horses.

All intact vessels relaxed in response to acetylcholine (Table 3; Fig 3) with the biggest response (as a percentage of phenylephrine pre-contraction) in laminar arteries (>>laminar veins = facial skin arteries; P = 0.02). Maximal relaxation in response to acetylcholine in laminar arteries was consistently greater than baseline tension (i.e. > 100% relaxation).

**Table 3:** Response of intact vessels from healthy horses and horses with endocrinopathic laminitis (EL) to acetylcholine following pre-contraction with phenylephrine.

		Intact Laminar Arteries		Intact Laminar Veins		Intact Facial Skin Arteries	
		Healthy	EL	Healthy	EL	Healthy	EL
Number		6	6	6	6	6	6
**Acetylcholine**	**Maximum % Relaxation**	323.5 ± 94.1	90.8 ± 4.4*	129.4 ± 14.8	71.2 ± 4.1*	182.0 ± 40.7	91.4 ± 4.5*
	**-Log IC**_**50**_ **(pD**_**2**_**)**	7.4 ± 0.3	7.9 ± 0.2	7.6 ± 0.2	7.2 ± 0.1	7.6 ± 0.2	8.2 ± 0.2

Maximal relaxation expressed as a percentage of initial contraction and sensitivity expressed as—Log EC_50_ (pD_2_) are shown. Data are mean ± SEM. Data were compared by Student’s t-test or Mann Whitney U (healthy vs EL).

* P<0.05 and

** P<0.005 compared with healthy.

### Removal of the endothelium from vessels of healthy horses

**Fig 4:**
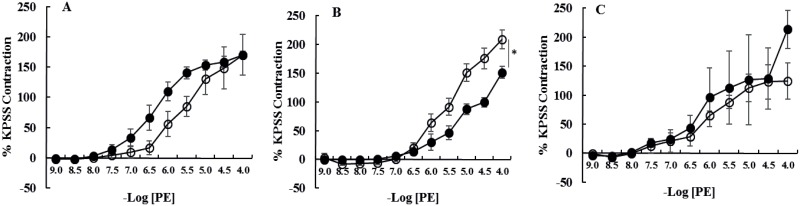
Cumulative concentration response curves to phenylephrine (PE) of denuded [A] laminar arteries, [B] laminar veins and [C] facial skin arteries from healthy horses (●, n = 6) and horses with endocrinopathic laminitis (○, n = 6). Data are expressed as a percentage of maximal contraction in response to high potassium saline (KPSS). Data are mean ± SEM. Curves were compared using two-way ANOVA. *P<0.05. There were no significant differences between healthy horses and those with EL.

**Fig 5:**
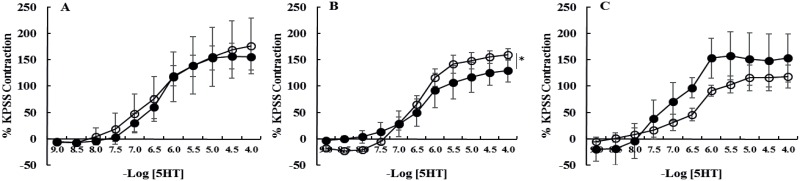
Cumulative concentration response curves to 5-hydroxytryptamine (5HT) of denuded [A] laminar arteries, [B] laminar veins and [C] facial skin arteries from healthy horses (●, n = 6) and horses with endocrinopathic laminitis (○, n = 6). Data are expressed as a percentage of maximal contraction in response to high potassium saline (KPSS). Data are mean ± SEM. Curves were compared using two-way ANOVA. *P<0.05. There were no significant differences between healthy horses and those with EL.

Removal of the endothelium abolished acetylcholine-induced relaxation in all vessels. Denuding did not alter the magnitude of contraction in response to phenylephrine or 5HT; however, the sensitivity of denuded laminar veins (but not laminar or facial skin arteries) to phenylephrine (P = 0.004) and 5HT (P = 0.0004) was decreased (Table 4, Figs 4 and 5).

**Table 4:** Response of denuded vessels from healthy horses and horses with endocrinopathic laminitis (EL) to vasoconstrictors high potassium saline (KPSS), phenylephrine (PE) and 5-hydroxytryptamine (5HT).

		Denuded Laminar Arteries		Denuded Laminar Veins		Denuded Facial Skin Arteries	
		Healthy	EL	Healthy	EL	Healthy	EL
Number		6	6	6	6	6	6
**KPSS**	**Emax mN/mm**	28.2 ± 4.1	26.1 ± 8.5	4.9 ± 1.7	8.8 ± 1.6*	19.5 ± 1.4	17.5 ± 2.7
**PE**	**Emax mN/mm**	31.7 ± 4.8	22.1 ± 3.2	5.3 ± 0.9	11.3 ± 1.4*	26.9 ± 14.4	21.0 ± 4.4
	**-Log EC**_**50**_ **(pD**_**2**_**)**	6.4 ± 0.2	5.9 ± 0.7*	5.4 ± 0.3	5.9 ± 0.4	6.4 ± 0.3	6.5 ± 0.6
**5HT**	**Emax mN/mm**	34.0 ± 3.8	23.7 ± 4.1	5.1 ± 1.9	10.4 ± 1.1*	29.7 ± 19.7	10.9 ± 3.5
	**-Log EC**_**50**_ **(pD**_**2**_**)**	6.5 ± 0.1	6.4 ± 0.7	6.4 ± 0.1	6.0 ± 0.3	7.0 ± 0.5	6.9 ± 0.7

Responses are given as absolute values (Emax mN/mm). Sensitivity is expressed as—Log EC50 (pD2). Data are mean ± SEM. Data were compared by Student’s t-test or Mann Whitney U (healthy vs EL)

* P<0.05.

### Vascular function in healthy horses compared to those with endocrinopathic laminitis

In comparison with healthy controls, acetylcholine-induced relaxation (Emax) was dramatically reduced in all vessels from horses with EL (Table 3; Fig 2). In addition, contractile responses (Emax mN/mm) to phenylephrine (P = 0.005) and 5HT (P = 0.0007) were increased in laminar veins from horses with EL (Table 2, Figs 1 and 2). Sensitivity to phenylephrine was reduced in laminar arteries (P = 0.006) and laminar veins (P = 0.009) from horses with EL (Table 2, Figs 1 and 2), this was an endothelium-independent effect in laminar arteries but not veins in which sensitivity was not significantly different when the veins were denuded.

## Discussion

The work presented here demonstrates that horses with endocrinopathic laminitis (EL) have vascular dysfunction manifest as blunting of endothelium-dependent vasodilation. This dysfunction is present in both the laminar vascular bed and distant facial skin arteries. In addition, laminar veins of EL horses showed further dysfunction with increased contractile responses to phenylephrine and 5HT, accompanied by a decrease in sensitivity to phenylephrine.

The vascular pathology associated with laminitis has been of interest for many years. Although there are reports of vascular dysfunction following experimentally induced inflammatory laminitis [38] there are no data pertaining to vascular dysfunction, local or systemic, in horses with EL, most likely due to the difficulties in obtaining samples from clinical cases. To assess vasoconstrictor function we used phenylephrine, an alpha-adrenoceptor agonist which produces stable and reliable concentration-dependent contractions of physiological relevance. Moreover, in treatment of laminitis alpha-adrenoceptor antagonists (e.g. acepromazine) are used clinically in an attempt to induce vasodilation [39]; phenylephrine is, therefore, also of pharmacological relevance in this disease. All three vessel types contracted in response to phenylephrine with both laminar and facial skin arteries having larger contractions than laminar veins. 5HT was used as an alternative contractile agonist in this study and elicited reproducible contractions of a similar magnitude to those induced by phenylephrine in all vessels. The response of equine vessels to 5HT has been a source of interest as both large and small equine veins are more sensitive to its effects than arteries [33, 40]. This has been proposed as a possible contributing factor to venous dysfunction in inflammatory laminitis in which circulating 5HT concentrations are elevated [38, 40], although elevated 5HT concentrations have not been demonstrated in EL. This differential sensitivity was replicated in our study. This differential sensitvity was endothelium-dependent consistent with other studies showing the response to 5HT is reliant on endothelial-derived nitric oxide [41]. Interestingly, while 5HT has been found to be significantly more potent in the larger palmer digital vessels compared to large facial arteries [40] there was no difference in sensitivity to this agent between small laminar arteries and small facial skin arteries.

Acetylcholine is commonly used in vascular function studies to induce endothelium-dependent relaxation via nitric oxide release [42]. As in other species, acetylcholine-mediated relaxation was endothelium-dependent in all vessels in this study. In contrast to the only other report of equine laminar vessel response to acetylcholine [43], laminar arteries in this study relaxed to greater than 200% of baseline tension in healthy horses. This is an unusual response in an isometric system, in which vessels do not normally develop tone unless stimulated, and is rarely observed in rodent vessels [42]. Some resistance vessels such as cerebral arteries exhibit myogenic tone in isometric systems [44], contracting in response to initial stretching without the addition of vasoconstrictors. The vessels in this study did not contract in response to application of baseline tension, indicating that myogenic tone did not develop. However, their response to acetylcholine implies that some degree of intrinsic tone is present in these vessels prior to application of baseline tension [45]. Intrinsic tone is normally a pathological response of vessels to high pressures; for example, in the context of pulmonary or renal hypertension [45]. The development of intrinsic tone in disease is usually accounted for by alterations in the mechanisms sensing or transducing a pressure stimulus, changes in shear wall stress or impaired endothelial function [46]. It is possible that laminar vessels have intrinsic tone as a normal physiological response to the much greater pressures they encounter than equivalent vessels in other species (capillary pressure in the standing horse’s hoof is approximately 35-50mmHg, compared to 20mmHg in the human foot and 15mmHg in the canine hind paw) [47–49]. However, given that intrinsic tone appears also to be a feature of facial skin arteries it is unlikely to be explained by elevated perfusion pressure and may be a species-specific finding. It is important to note that, even though vessels from EL horses relaxed significantly less than those from healthy horses, the arteries still relaxed by 100% whereas the mean relaxation of the veins was only 70%. This most likely reflects endothelial dysfunction of the veins but, in the arteries, a loss of intrinsic tone may also play a role.

Inflammatory laminitis, induced with administration of black walnut extract, is known to result in failure of larger conduit vessels of the equine limb to adequately dilate in response to acetylcholine but the mechanism is unknown [50]. These experimental models are comparable to sepsis and multi organ failure in humans in which endothelial cell dysfunction is an important feature [51]. This is the first study to demonstrate similar vascular dysfunction in EL though systemic hypertension has been reported in ponies with insulin resistance [52] but whether this is due to vascular dysfunction is unknown. EL is associated with either EMS or PPID; these conditions have several features in common which may explain the evident vascular dysfunction. Both diseases are associated with insulin dysregulation, abnormal adiposity and probable cortisol dysregulation [53].

In this study all of the horses with EL had insulin dysregulation manifest as fasting hyperinsulinaemia. Hyperinsulinaemia can result in endothelial cell dysfunction, specifically blunting vasodilatory responses, as well as endothelial resistance to insulin- induced enhancement of vasodilation [54]. Insulin resistance results in a downregulation of the PI3kinase pathway with a subsequent reduction in NO production whilst the compensatory hyperinsulinemia causes excessive stimulation of the MAP Kinase pathway and production of ET-1 and reactive oxygen species in humans [55]. *Ex vivo* models of equine vessels incubated in high concentrations of insulin have shown that a similar phenomenon may also occur in horses [56] and this pathophysiological process may contribute to the endothelial dysfunction evident in this study.

The role of cortisol dysregulation in vascular function is less well defined as it is often difficult to distinguish the effects of hypercortisolaemia from the accompanying metabolic syndrome in humans. Human Cushing’s disease patients treated with surgery remain at high risk of cardiovascular disease years after the hypercortisolaemia has abated, most probably due to the persistence of features of metabolic syndrome [57]. However, human Cushing’s patients also show increased insulin-stimulated endothelin release which is not a feature of metabolic syndrome alone [58]. While EMS and PPID are not associated with hypercortisolaemia there is evidence of peripheral cortisol dysfunction in chronic laminitis which deserves further study [59]. Cortisol inhibits endothelial-dependent cholinergic vasodilation in humans most likely through an inhibition of NO synthesis [17], reduces the bioavailability of NO in vessels [60], inhibits tetrahydrobiopterin [61, 62], the cofactor necessary for maximal activity of nitric oxide synthase and potentiates the effects of vasoconstrictors [18, 63].

Expanding adipose tissue adopts a pro-inflammatory phenotype accompanied by increased production of reactive oxygen species. Obesity is associated with an uncoupling of eNOS, such that it produces superoxide ion (O2-) rather than NO, an event known to precede the establishment of other obesity co-morbidities [64]. A pro-inflammatory phenotype has been demonstrated in horses with insulin resistance and obesity [65, 66] but it has yet to be ascertained whether this is accompanied by dysregulation of NO.

Laminar veins showed more evidence of dysfunction in EL horses in this study compared with laminar and facial skin arteries. Veins are rarely studied in rodent models of vascular dysfunction or in human disease but have been shown to have altered function in experimentally-induced inflammatory models of laminitis in the horse [38, 67]. There are differences in the mechanism of response to vasoconstrictors between laminar arteries and veins. For example unlike arteries, laminar veins still contract in response to phenylephrine and 5HT when there is no extracellular calcium present [68]. Such mechanistic differences may go some way to explain the apparent preferential dysfunction of veins in disease. The venous return from the hoof relies on adequate relaxation of the veins and the pump action of the hoof hitting the ground [69]. Adequate venous return is essential to prevent elevation in capillary pressure and oedema. Reduced relaxation of the veins in disease may be of equal, if not more, importance than arterial dysfunction in the pathophysiology of EL and warrants further investigation.

It is important to note that the dysfunction identified in this study could be a cause or a consequence of disease. Failure of the vessels within the hoof to adequately dilate will have significant consequences, potentially reducing overall blood flow and reducing venous return. Such abnormalities may lead to increased capillary pressure and oedema and hypoxia of the laminar tissue. Equally systemic endothelial dysfunction may render these animals susceptible to other vascular and microvascular abnormalities such as retinal vascular lesions [70] not previously investigated in this population. Clinical vascular markers of endothelial dysfunction such as flow mediated dilatation may not be readily transferrable to equine medicine. However, plasma biomarkers of endothelial dysfunction, such as asymmetrical dimethylarginine and oxidized LDL [71], would be an invaluable clinical tool for determining laminitic risk and monitoring response to treatment. It is plausible that abrogating endothelial dysfunction may be beneficial in horses with EL in order to prevent or manage laminitis. Pharmacological interventions aimed at restoring blood flow to the hoof by alpha adrenoceptor antagonism (acepromazine, domperidone) or beta-adrenoceptor agonists (isoxsuprine hydrochloride) have been used clinically with varying success [39, 72], possibly reflecting our limited understanding of vascular dysfunction in laminitis. Given the high prevalence of this disorder and the clinical consequences there is still an urgent unmet clinical need in this area of veterinary medicine.

In conclusion, this study has shown endothelial dysfunction associated with EL affecting both laminar vessels and distant facial skin arteries. The endothelium may be a target for the treatment or diagnosis of EL.
